# The role of *Atg5* gene in tumorigenesis under autophagy deficiency conditions

**DOI:** 10.1002/kjm2.12853

**Published:** 2024-06-03

**Authors:** Hsiao‐Sheng Liu, Yin‐Ping Wang, Pei‐Wen Lin, Man‐Ling Chu, Sheng‐Hui Lan, Shan‐Ying Wu, Ying‐Ray Lee, Hong‐Yi Chang

**Affiliations:** ^1^ Department of Microbiology and Immunology, College of Medicine National Cheng Kung University Tainan Taiwan; ^2^ Tropical Medicine College of Medicine Kaohsiung Medical University Kaohsiung Taiwan; ^3^ Center for Cancer Research, College of Medicine Kaohsiung Medical University Kaohsiung Taiwan; ^4^ Teaching and Research Center, Kaohsiung Municipal Siaogang Hospital, Kaohsiung Medical University Hospital Kaohsiung Medial University Kaohsiung Taiwan; ^5^ Department of Life Sciences and Institute of Genome Sciences National Yang Ming Chiao Tung University Taipei Taiwan; ^6^ Cancer Progression Research Center National Yang Ming Chiao Tung University Taipei Taiwan; ^7^ Department of Microbiology and Immunology, College of Medicine Taipei Medical University Taipei Taiwan; ^8^ Graduate Institute of Medical Sciences, College of Medicine Taipei Medical University Taipei Taiwan; ^9^ Department of Microbiology and Immunology, School of Medicine, College of Medicine Kaohsiung Medical University Kaohsiung Taiwan; ^10^ Department of Anatomy, School of Medicine, College of Medicine Kaohsiung Medical University Kaohsiung Taiwan; ^11^ Department of Medical Research Kaohsiung Medical University Hospital Kaohsiung Taiwan

**Keywords:** autophagy, autophagy‐related gene 5 (*Atg5*), tumorigenesis

## Abstract

Autophagy is a self‐recycling machinery to maintain cellular homeostasis by degrading harmful materials in the cell. Autophagy‐related gene 5 (*Atg5*) is required for autophagosome maturation. However, the role of Atg5 in tumorigenesis under autophagy deficient conditions remains unclear. This study focused on the autophagy‐independent role of Atg5 and the underlying mechanism in tumorigenesis. We demonstrated that knockout of autophagy‐related genes including *Atg5*, *Atg7*, *Atg9*, and *p62* in mouse embryonic fibroblast (MEF) cells consistently decreased cell proliferation and motility, implying that autophagy is required to maintain diverse cellular functions. An Atg7 knockout MEF (Atg7^−/−^ MEF) cell line representing deprivation of autophagy function was used to clarify the role of Atg5 transgene in tumorigenesis. We found that Atg5‐overexpressed Atg7^−/‐^MEF (clone A) showed increased cell proliferation, colony formation, and migration under autophagy deficient conditions. Accordingly, rescuing the autophagy deficiency of clone A by overexpression of *Atg7* gene shifts the role of Atg5 from pro‐tumor to anti‐tumor status, indicating the dual role of Atg5 in tumorigenesis. Notably, the xenograft mouse model showed that clone A of Atg5‐overexpressed Atg7^−/−^ MEF cells induced temporal tumor formation, but could not prolong further tumor growth. Finally, biomechanical analysis disclosed increased Wnt5a secretion and p‐JNK expression along with decreased β‐catenin expression. In summary, Atg5 functions as a tumor suppressor to protect the cell under normal conditions. In contrast, Atg5 shifts to a pro‐tumor status under autophagy deprivation conditions.

## 1 INTRODUCTION

Autophagy is an intracellular self‐degradative process that plays a critical role in response to various cellular stresses for cell survival.^1^ Normal cells keep baseline levels of autophagy to maintain cellular homeostasis.^2^ During starvation or stress conditions, autophagy is upregulated to protect cells by degradation of cytosolic components, followed by recycling of nutrients.^1^ Autophagy begins with the encapsulation of dysfunctional organelles and cytoplasmic molecules into double‐membrane autophagosomes which then fuse with lysosomes for degradation.^3^ Therefore, failure to remove dysfunctional or aggregated proteins as well as damaged organelles through autophagy‐mediated degradation may lead to multiple pathological‐related diseases.^4^ Autophagy has been reported to have a tumor‐suppressive function, and manipulating autophagy may potentially prevent or inhibit tumorigenesis.^5^ Conversely, autophagy has also shown tumor‐promoting roles in the survival of malignant cancer cells under chemotherapy, hypoxic environment, anoikis, and metastatic conditions.^6^ For various diseases, autophagy plays an adaptive role in overcoming cellular stresses (e.g., nutrient starvation and hypoxia) in order to promote survival of tumor cells.^7^ Tumorigenesis is a multistep process that arises in a normal cell that undergoes cellular genome instability, loss of control of the cell cycle checkpoint, and abnormal activation of oncogene and/or tumor suppressor genes, leading to tissue invasion and distant metastasis.^8^ Taken together, the current evidence indicates that autophagy plays a dual role in tumorigenesis depending on cancer cell type, cancer stage, and the microenvironment.

The autophagic mechanism consists of four stages, namely, initiation, membrane elongation, maturation, and degradation,^9^ and the mechanism is tightly regulated by over 30 autophagy‐related gene (ATG)‐encoded proteins.^10^ These proteins are divided into five functional groups: the ULK kinase core complex, the autophagy‐specific class III phosphatidylinositol 3‐kinase (PI3K) complex, the ATG9A trafficking system, the ATG12 ubiquitin‐like conjugation system, and the LC3 ubiquitin‐like conjugation system.^11^ For example, Atg9 is required for pre‐autophagosomal structure (PAS) assembly, Atg5 and Atg7 are essential for autophagosome formation, and p62 is required for autophagic degradation.^11^ The ATG proteins are recruited hierarchically proximal to the vesicles and organize the PAS, which is essential for autophagosome formation. Autophagy plays a critical role in tumorigenesis. For example, Beclin 1 inhibited proliferation in human MCF‐7 breast cancer cells and decreased the tumorigenic potential in nude mice.^12^ Moreover, mutations of Atg genes were detected in gastric and colorectal cancers with microsatellite instability, implying that autophagy deficiency may lead to tumor development.^13^ In contrast, deletion of the *Atg5* or *Atg7* gene suppressed malignant cancer progression^14^ depending on the status of p53 in murine pancreatic tumor models.^15^ Furthermore, Atg5 has been shown to be a tumor suppressor in various cancers under normal autophagy conditions.^16^ Therefore, these findings imply that autophagy‐related protein is involved in tumor suppression.

We have reported that overexpression of Atg5 could suppress Ha‐*ras*‐induced tumor formation.^17^ This finding indicates that Atg5 plays a suppressive role in tumorigenesis under normal autophagy conditions. However, the role of Atg5 without the influence of autophagy and the underlying mechanism in tumorigenesis remains unclear. In this study, an autophagy‐deficient Atg7^−/‐^MEF (Atg7^−/−^ MEF) cell line was used as the cell line model to clarify the autophagy‐independent role of Atg5 in tumorigenesis. We further revealed the signaling pathway of Atg5 in tumorigenesis. In summary, we found that Atg5 plays a dual role in tumorigenesis depending on the autophagy status.

## 2 MATERIALS AND METHODS

### 2.1 Cell lines

Wild‐type mouse embryonic fibroblasts (WT‐MEF), Atg5 knockout MEF (Atg5^−/−^ MEF), Atg7 knockout MEF (Atg7^−/−^ MEF), Atg9 knockout MEF (Atg9^−/−^ MEF), and p62 knockout MEF (p62^−/−^ MEF) were kindly provided by Dr. Noboru Mizushima (University of Tokyo, Japan). Atg7^−/−^ MEF cells stably expressing Atg5 transgene were established by calcium phosphate transfection followed by selection with blasticidin (Sigma, MO, USA) for 1 month. All cells were grown in Dulbecco's modified Eagle's medium (Invitrogen, NY, USA) supplemented with 10% fetal bovine serum (GeneDirex, NV, USA) and penicillin/streptomycin (Sigma) at 37°C under 5% CO_2_ conditions.

### 2.2 Plasmids, transient transfection, and calcium phosphate transfection

Plasmids pEGFP‐Atg5 was kindly provided by Dr N. Mizushima, pSF‐Atg7 was a gift from Dr T. Yoshimori. Transient transfection of plasmid DNAs was performed by Lipofectamine™ 2000 according to the manufacturer's instructions (Invitrogen, NY, USA). Cells were harvested 48 h after transfection for the following experiments. Overexpression of Atg5 in Atg7^−/−^ MEF stable cell lines was established by calcium phosphate transfection. Cells (1 × 10^6^) were seeded in a 10‐cm petri dish 1 day before transfection. DNA (25 μg) was resuspended with 0.5 mL CaCl_2_ together with 0.5 mL of HEPES solution. DNA mixture was incubated at RT for 20 min and then added into the medium.

### 2.3 MTT assay

Cells (2.0 × 10^3^/well) were seeded in a 96‐well plate in 6 replicates. After incubation for 24, 48, and 72 h, 10 μL MTT [3‐(4, 5‐dimethylthiazolyl‐2)‐2,5‐diphenyltetrazolium bromide] reagent was added to each well and incubated for 3 h until purple precipitation was visible. The medium was then replaced with 100 μL DMSO and thoroughly mixed. Absorbance was recorded at 490 nm wavelength using an iMark™ Microplate Absorbance Reader (Bio‐Rad, CA, USA).

### 2.4 Cell viability assay

Cells were trypsinized and washed by PBS. Pellets were collected after centrifugation at 1500 rpm for 5 min and then kept on ice, followed by 0.04 mg/mL propidium iodide (PI) (Sigma) staining. The cell viability was determined by FACScan™ (BD Immunocytometry Systems, NJ, USA). Data analysis was conducted by examining plots of forward scatter versus red propidium iodide (PI) cell fluorescence (Log PI), facilitating the differentiation between viable cells and those that have taken up PI indicating cell death. Cells exhibiting PI fluorescence were identified as dead cells, with quantification performed by analyzing the dot plots. The results were analyzed using the GraphPad Prism software.

### 2.5 BrdU incorporation assay

Cells (1.5 × 10^5^) were seeded in a 6‐well plate. After 48 h incubation, cells were grown in the medium containing 0.01 g/mL bromodeoxyuridine (BrdU) (Sigma) for 20 min, fixed in acidic ethanol at −20°C for 10 min, and then incubated with 2 N HCl for 10 min. Anti‐BrdU polyclonal antibody (GE Healthcare, BM, UK) was added at 1:100 dilution and incubated at 4°C overnight. The cells were then viewed under a fluorescent microscope (DP 72, Olympus, PA, USA).

### 2.6 Western blotting

Proteins of cell lysates were collected by centrifugation at 18,000 × *g* for 20 min at 4°C after lysing the cells in RIPA buffer containing Na_3_SVO_4_, EGTA, PMSF, aprotinin, leupeptin, and EDTA. Proteins of the conditioned medium were collected using Amicon™ Ultra‐4 Centrifugal Filter Units (EMD Millipore, MA, USA) according to the manufacturer's instructions. Protein concentration was measured with a Pierce™ Coomassie (Bradford) Protein Assay Kit (Thermo Scientific, MA, USA). Total proteins were denatured and then separated by 12% SDS‐polyacrylamide gel, transferred to polyvinylidene difluoride membranes (EMD Millipore), and then incubated in 2% (v/v) fetal bovine serum. The antibodies used were: β‐actin (A5441, Sigma), LC3 (PM036, MBL), Atg5 (ab108327, Abcam), Atg7 (ab133528, Abcam), p62 (PM045, MBL), β‐catenin (GTX101435, GeneTex), Wnt5a (GTX100618, GeneTex), phospho‐SAPK/JNK (#4668S, Cell Signaling), and SAPK/JNK (#9252, Cell Signaling).

### 2.7 Cell migration assay

Transwell™ Permeable Supports (Corning Costar Corp., MA, USA) were used to evaluate the cell migration. The complete medium with 10% FBS was added into the lower chamber. Cells (1 × 10^5^ cells) suspended in serum‐free medium were seeded in the upper chamber and incubated for 6 h at 37°C. The non‐migrated cells in the upper chamber were carefully removed by cotton swabs and migrated cells were fixed with 1% formaldehyde and stained with 0.1% crystal violet. Results were counted under a light microscope in 10 randomly selected fields.

### 2.8 Xenograft tumor mouse model

The 4‐week‐old female NOD/SCID mice were purchased from the Laboratory Animal Center of National Cheng Kung University, College of Medicine, Tainan, Taiwan. Six mice were injected subcutaneously (s.c.) with vector control, clone A, and M5R cells (1 × 10^7^ cells/100 μL) in the left and right flanks. Tumor formation was observed 3 days after injection. Tumor size was measured according to the formula: volume (mm^3^) = (length × width^2^)/2. The animal experiments complied with Taiwan's Animal Protection Act, and the protocol of the xenograft mouse experiment was approved by the Laboratory Animal Care and Use Committee of the National Cheng Kung University (Approval No. 99079). The percentage of tumor formation was calculated by final tumor numbers divided by overall tumor cell inoculation sites × 100‰.

### 2.9 Statistical analysis

Data are presented as the mean ± standard deviation (SD). The *p*‐value was calculated using the Student's *t*‐test. The statistical analysis was performed by GraphPad Prism software 6.0. *P* ≤ 0.05 was considered statically significant: *P ≤ 0.05, **P ≤ 0.01, and ***P ≤ 0.001 and ns for no significance.

## 3 RESULTS

### 3.1 MEF cell lines harboring knockout of autophagic genes showed suppression of cell growth and migration

**FIGURE 1:**
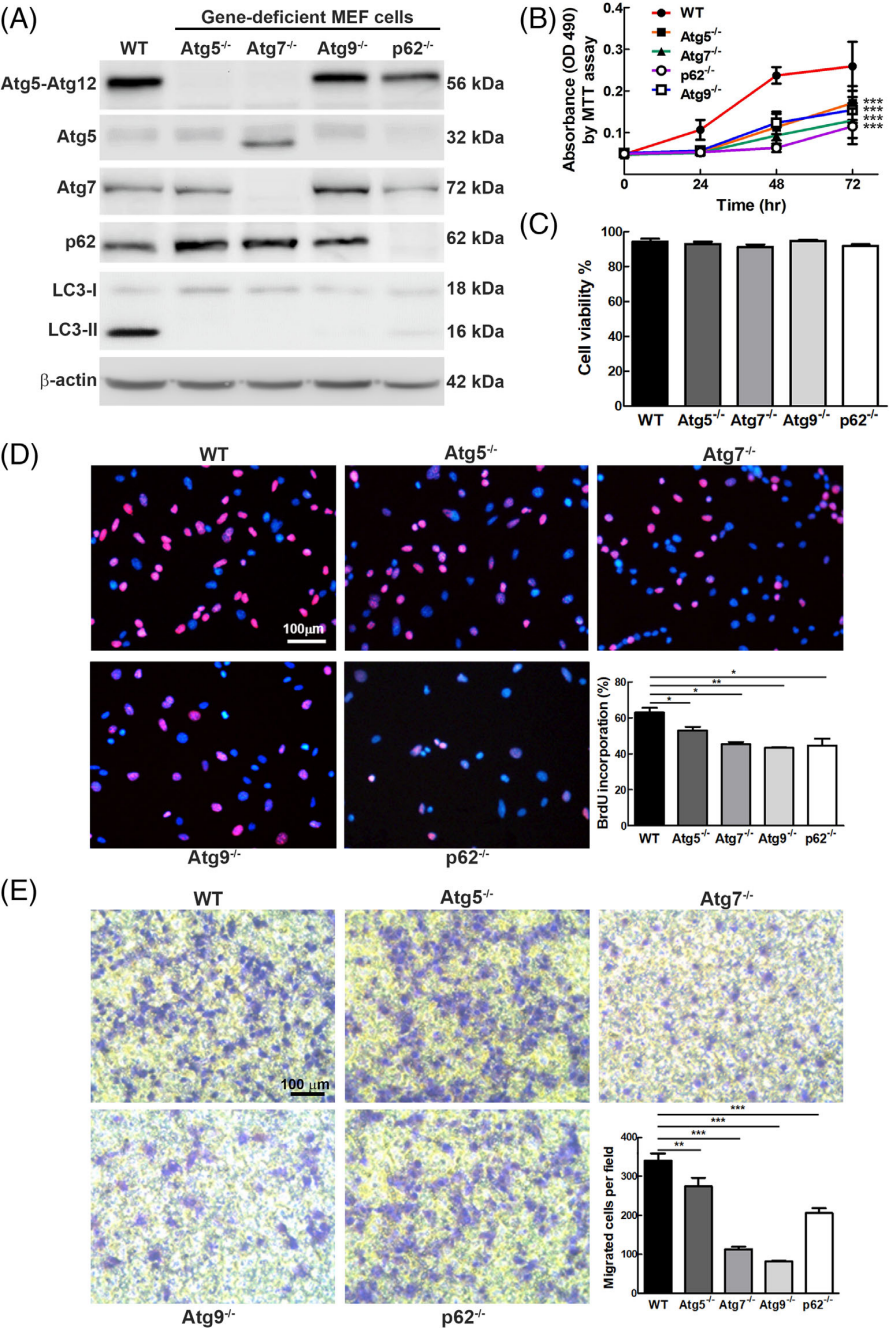
MEF cell lines harboring knockout of autophagy‐related genes showed suppression of cell growth and migration. (A) Protein levels of Atg5, Atg7, Atg9, p62, and LC3 in 4 MEF knockout cell lines and wild‐type MEF cells were analyzed using specific antibodies by Western blotting. β‐Actin was used as the internal control. (B) Proliferation of the five MEF stable cell lines in (A) was measured by MTT analysis at 24, 48, and 72 h. (C) Cell viability of the five MEF cell lines was determined by staining the cells with propidium iodide (PI, 0.04 mg/mL) followed by flow cytometry analysis. (D) DNA synthesis of the five MEF cell lines was evaluated by BrdU incorporation assay. Cells treated with BrdU (0.01 g/mL) for 20 min followed by anti‐BrdU antibody and Hoechst labeling of the proliferating cells and nuclei, respectively. Purple color represents proliferating cell, which was counted under three random fields under a fluorescent microscope at 200× followed by quantification. (E) Cell motility of the five MEF stable cell lines was determined by Transwell assay. Cells (1 × 10^5^ cells) were seeded on the upper chamber for 6 h, and migrated cells were stained with 0.1% crystal violet and counted under three random fields under a light microscope at 100× followed by quantification. Data represented as mean ± SD and error bars represent SD. The differences between autophagy gene knockout and WT MEF cell lines were statistically analyzed by Student's *t*‐test. *P*‐values less than 0.05 were considered significant. *P < 0.05, **P < 0.01, or ***P < 0.001.

Because multiple Atg gene knockout mice models have been used to study the relationship between autophagy and tumorigenesis. The wild‐type mouse embryonic fibroblast (WT‐MEF) was chosen as the parental cell line to clarify the potential role of autophagy in cell growth, migration, and tumorigenesis. WT‐MEF derivative cell lines harboring knockout of four essential Atg genes, (*Atg5, Atg7, Atg9*, and *p62*) were generated and designated as: Atg5^−/−^ MEF, Atg7^−/−^ MEF, Atg9^−/−^ MEF and p62^−/−^ MEF. These Atg genes participate in the autophagy process, including vesicle nucleation (Atg9), vesicle elongation (Atg5 and Atg7), and vesicle degradation (p62). We reveal that Atg5‐Atg12, Atg7, and p62 protein expression was significantly decreased in the MEF derivative of Atg5^−/−^, Atg7^−/−^ and p62^−/−^ by Western blotting (Figure 1A). Notably, we found that Atg5 monomer was trivially detected in WT‐MEF, it is prone to be conjugated with Atg12 (Atg5‐Atg12) for autophagy progression (Figure 1A and Supplementary Figure S1, lane 1). In Atg5^−/−^ MEF cells, the Atg5‐Atg12 levels become undetectable compared to WT‐MEF (Figure 1A and Supplementary Figure S1, lane 2), implying that the *Atg5* gene was abolished. We further demonstrated that the exon 6 to exon 11 region of *Atg9* gene was abolished by replacing with PGK + neo gene in Atg9^−/−^ MEF cells by PCR analysis (Supplementary Figure S2). The LC3‐II expression levels representing autophagy activity become undetectable in the four Atg‐knockout (Atg‐KO) cell lines compared to WT‐MEF cells (Figure 1A and Supplementary Figure S1), indicating autophagy deficiency in these four Atg‐KO cell lines.

Functional analysis reveals that the cell proliferation of the four Atg KO cell lines was decreased at 48 and 72 h compared to that of WT MEF cells by MTT assay (Figure 1B, *p* < 0.001). Similarly, colorectal cancer SW480 and gastric cancer AGS cell lines also showed decreased cell survival rates by shRNA silencing Atg5 expression or CRISPR knockout Atg5 gene (Supplementary Figures S3 and S4). These data indicate that abolishing essential autophagy genes including Atg5 under normal conditions decreased cell number and knockout Atg5 gene showed more significant suppression of cell proliferation compared to silencing Atg5 gene in colon cancer cells. Because WT‐MEF similar to NIH3T3 cells with the unique characteristics of high transfection efficiency and potential tumorigenicity, which are widely used for defining oncogenic genes.^18^ Therefore, MEF and its derivate are used in the following experiments. Despite abolishing Atg5, 7, 9 and p62 genes cause decreased cell numbers (Figure 1B), the cell viability of these four Atg‐KO cell lines was no significant difference compared to that of WT‐MEF cells, as demonstrated by flow cytometry analysis (Figure 1C). We further conducted the BrdU incorporation assay to evaluate the DNA synthesis of these cell lines. Our data showed that the BrdU incorporation ratio of these four Atg‐KO MEF cell lines was significantly decreased compared to WT‐MEF cells (Figure 1D). These data imply that the reduced growth kinetics of the four Atg‐KO cell lines were caused by decreased DNA synthesis, not increased cell death (Figure 1C, D). We further clarified the motility of the four Atg‐KO MEF cell lines. Our data showed that these four autophagy‐deficient cell lines showed various degrees of decrease in cell migration compared to WT‐MEF cells (Figure 1E). In summary, dysfunctional autophagy leads to decreased cell proliferation and migration through decreased DNA synthesis, without affecting the cell viability.

### 3.2 Proliferation, migration, and colony formation of Atg7^−/−^ MEF cells overexpressing *Atg5* gene were increased

**FIGURE 2:**
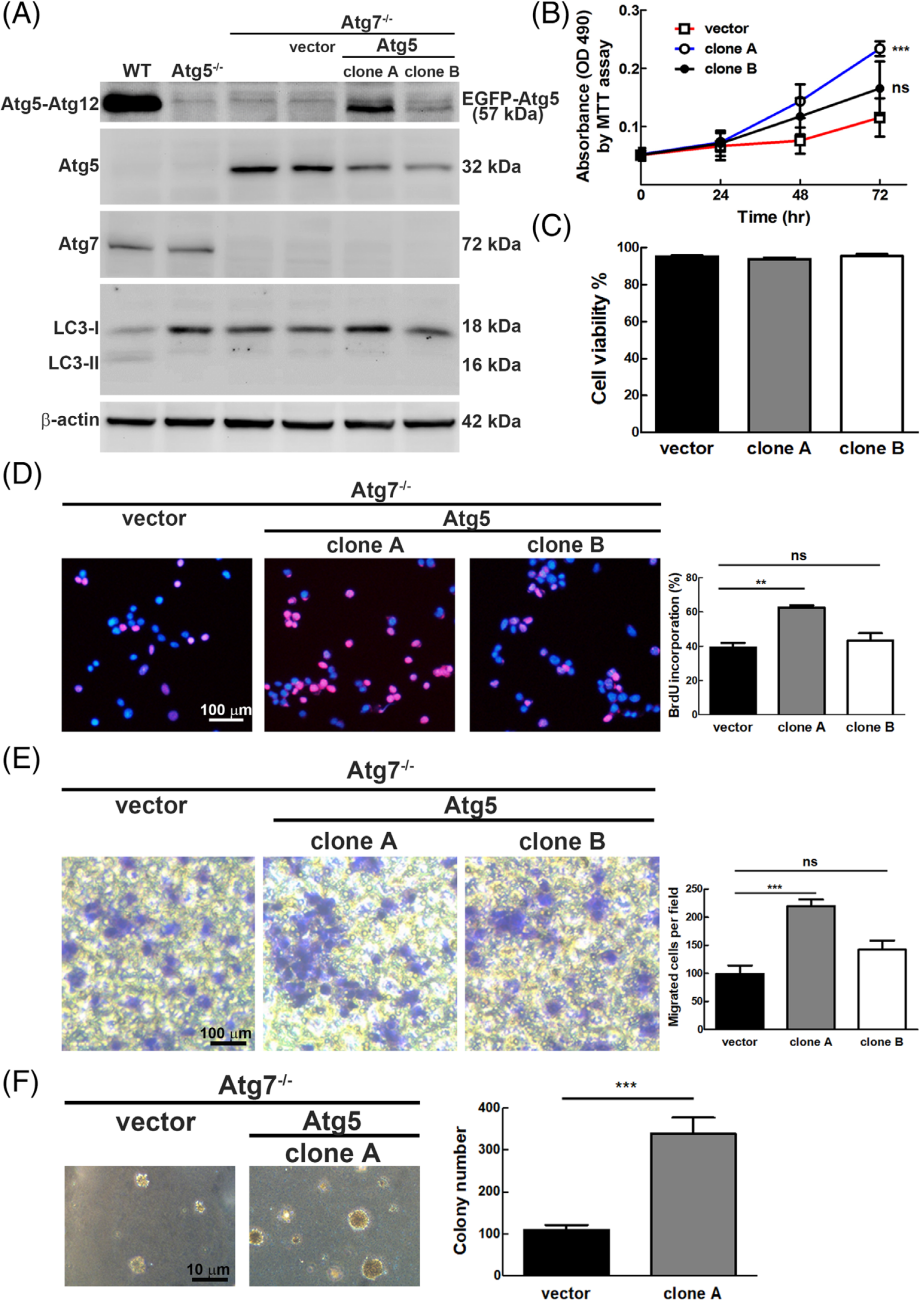
Proliferation, migration and colony formation of Atg7^−/−^ MEF cells overexpressing exogenous *Atg5* gene were increased. (A) Protein levels of Atg7, Atg5, and LC3 were evaluated by Western blotting of two Atg5‐overexpressed Atg7^−/−^ MEF stable clones (clone A and B) and the vector control (vector). The levels of Atg7, Atg5, LC3 were determined using specific antibodies for Western blotting analysis. (B) Proliferation of the three MEF cell lines in (A) was measured by MTT analysis at 0, 24, 48, and 72 h. (C) Cell viability of the three MEF cell lines was determined by staining the cells with PI (0.04 mg/mL) followed by flow cytometry analysis. (D) DNA synthesis of the three MEF cell lines was evaluated by BrdU incorporation assay (the same procedure as in Figure 1D). (E) Cell motility of clone A, clone B, and vector control cells were determined by Transwell™ assay. The procedure was the same as in Figure 1D. (F) Clone A of Atg5‐overexpressed Atg7^−/−^ MEF cells was selected for anchorage‐independent colony formation assay. Cells were plated on 0.6% soft agar in a 6‐well tray for 14 days, and colonies were counted at 9× magnification. Left panel, the morphology of colonies in the soft agar was observed under a light microscope at 40× magnification. Right panel, colonies were counted at 9× magnification, and 5 fields were randomly counted. Data represented as mean ± SD and error bars represent SD. The differences between autophagy gene knockout and WT MEF cell lines were statistically analyzed by Student's *t*‐test. *P*‐values less than 0.05 were considered significant. *P < 0.05, **P < 0.01, or ***P < 0.001.

We previously reported that the tumor formation of MEF‐Atg5(−/−)‐Ha‐*ras* ^val12^ cells was decreased after overexpression of HA‐tagged Atg5 transgene in a xenograft mouse model, indicating that Atg5 plays a tumor‐suppressive role in tumor formation under normal autophagy conditions.^17^ However, the role of Atg5 without functional autophagy in tumorigenesis remains unclear. To elucidate the potential role of Atg5 in tumorigenesis without the effect of autophagy, we established stable cell lines overexpressing Atg5 transgene under autophagy deficient background by knockout Atg7 gene (Atg7^−/−^ MEF). Briefly, the Atg7^−/−^ MEF cells were co‐transfected with pEGFP‐Atg5 or vector plasmid (pEGFP‐C1) together with pTRE2‐BSD (blasticidin) by the calcium phosphate method, followed by the selection of blasticidin resistant clones for 2 weeks. We isolated two clones expressing high and low levels of conjugated Atg5‐Atg12 protein in Atg7^−/−^ MEF cells designed as clone A and clone B, respectively (Figure 2A, lane 5 and 6). Our results showed that no LC3‐II expression was detected in Atg7^−/−^ MEF parental and derivative cells expressing either endogenous Atg5 monomer (32 kDa) or Atg5‐Atg12 together with EGFP‐Atg5 (57 kDa) (Figure 2A, lane 2 to 6). These two autophagy‐deficient clones overexpressing Atg5 were used to clarify the autophagy‐independent role of Atg5 in tumorigenesis. Initially, we reveal the role of Atg5 in cell growth and viability by MTT assay and flow cytometry. Clone A showed the highest cell proliferation rate followed by clone B compared to the vector control at 72 h (Figure 2B). In contrast, clone A and B of Atg5‐overexpressed Atg7^−/−^ MEF cells showed no significant difference in cell viability compared to the vector control (Figure 2C), which is similar to the result of Figure 1C. We further examined the DNA synthesis rate of the cells by BrdU incorporation assay. We found that only clone A expressing higher GFP‐Atg5 (Figure 2A, lane 4) showed a significantly higher BrdU incorporation rate compared to clone B and the vector control (Figure 2D), which is consistent with the trend of cell proliferation in Figure 2B. It implies that the high cell proliferation of clone A was caused by an increased DNA synthesis rate.

Furthermore, we clarified the migration of Atg5‐overexpressed Atg7^−/−^ MEF cell lines by Transwell™ assay. Similarly, the cell migration of clone A was the highest compared to clone B and the vector control (Figure 2E). Altogether, overexpression of Atg5 transgene under autophagy deficient conditions significantly increased cell proliferation and migration of MEF cells. Anchorage‐independent colony formation is a hallmark of tumor formation in vitro.^19^ Therefore, we conducted a soft agar assay to examine colony formation using clone A. Our results showed that for clone A both of the number and size of the colony were significantly increased compared to the vector control (Figure 2F), indicating that Atg5 expression increased colony formation under autophagy‐deficient conditions.

### 3.3 Overexpression of Atg5 in Atg7^−/−^ MEF cells (clone A) transiently promoted tumor formation in a xenograft NOD/SCID mouse model

**FIGURE 3:**
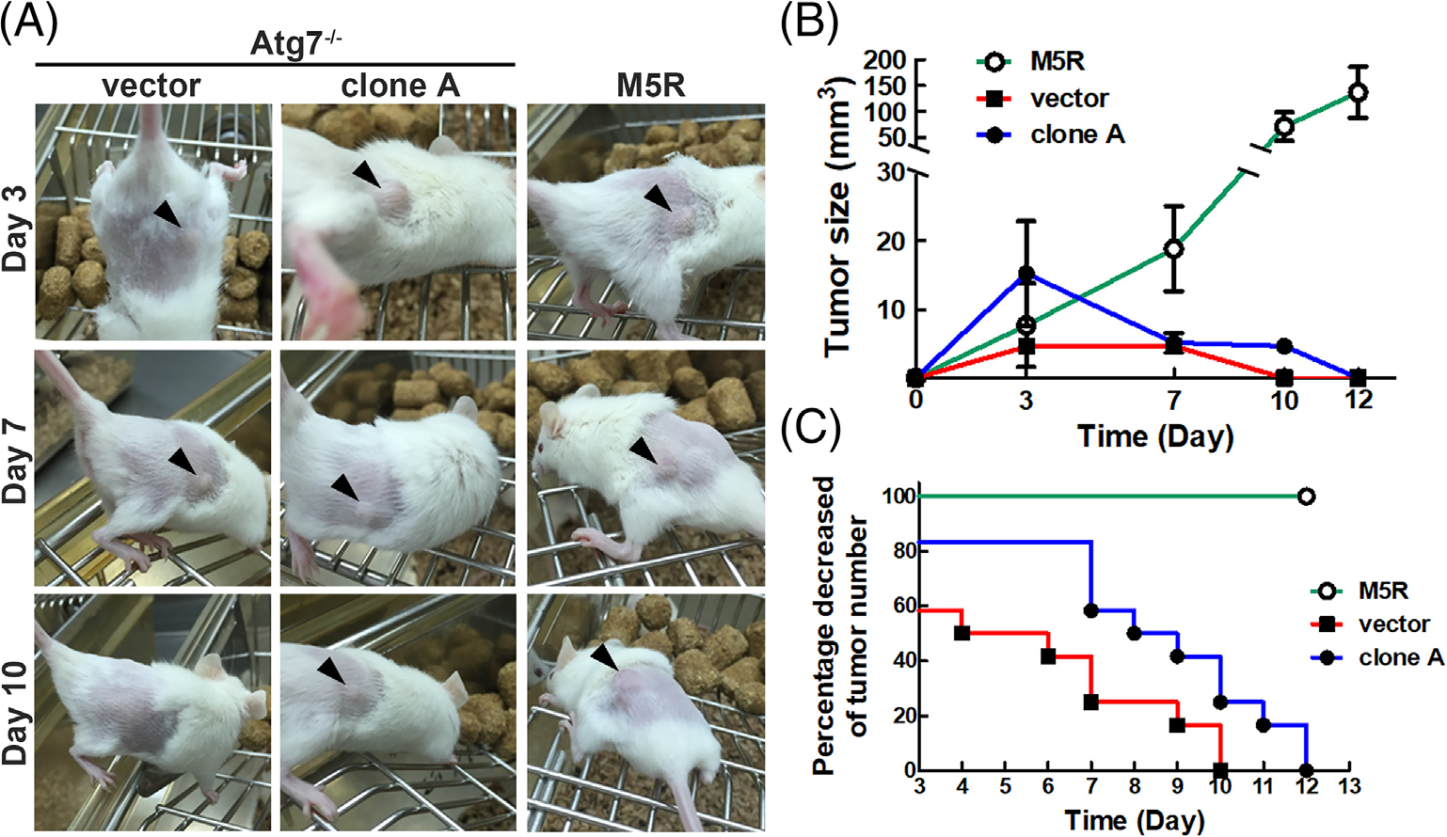
Overexpression of Atg5 in Atg7^−/−^ MEF cells (clone A) transiently promoted tumor formation in xenograft NOD/SCID mouse model. Clone A (Overexpression of Atg5 in Atg7^−/−^ MEF), vector control, and MEF‐Atg5^−/−^/Ha‐*Ras* ^val12^ (M5R) cells were subcutaneously inoculated into the immunocompromised NOD/SCID mice. (A) Tumor size in the mice was measured at days 3, 5, 7, and 12 (p.i.). Arrowhead points to the tumor of the mice. (B) The kinetics of tumor volume in three groups was measured at different time points (p.i.). (C) Percentage of tumor formation is calculated by final tumor number divided by overall sites of cancer cell inoculation in each group. Error bars represent SD.

Finally, we clarified whether Atg5 overexpression in autophagy‐deficient MEF cells (clone A) affects tumor formation in vivo. Clone A expressing the Atg5 transgene and vector control of Atg7^−/−^ MEF cells were subcutaneously injected into the flanks of four‐week‐old female NOD/SCID mice. Since MEF is an immortalized cell line, we used previously established stable MEF‐Atg5^−/−^‐Ha‐*ras* ^val12^ (M5R) cell line^17^ as the tumor formation positive control. Our results showed that inoculation of NOD/SCID mice with either clone A (Atg5‐overexpressed Atg7^−/−^ MEF) or M5R positive control cell line formed larger tumors compared to the vector control at day 3 post‐injection (p.i.) (Figure 3A, B). Tumor sizes of M5R cells were continuously increased from day 3 to day 12 (p.i.). Intriguingly, the tumor size of clone A reached the maximum at day 3 p.i., and then started to regress till day 12 p.i. (Figure 3A, B). Similarly, M5R positive control showed 100% tumor formation, and clone A showed a slower reduction of tumor number compared to the vector control group (Figure 3C). Taken together, our data imply that overexpression of Atg5 under autophagy‐deficient conditions transiently induced tumor formation followed by tumor regression in vivo.

### 3.4 Rescuing autophagy deficiency of clone A by overexpressing Atg7 suppressed cell proliferation and migration

**FIGURE 4:**
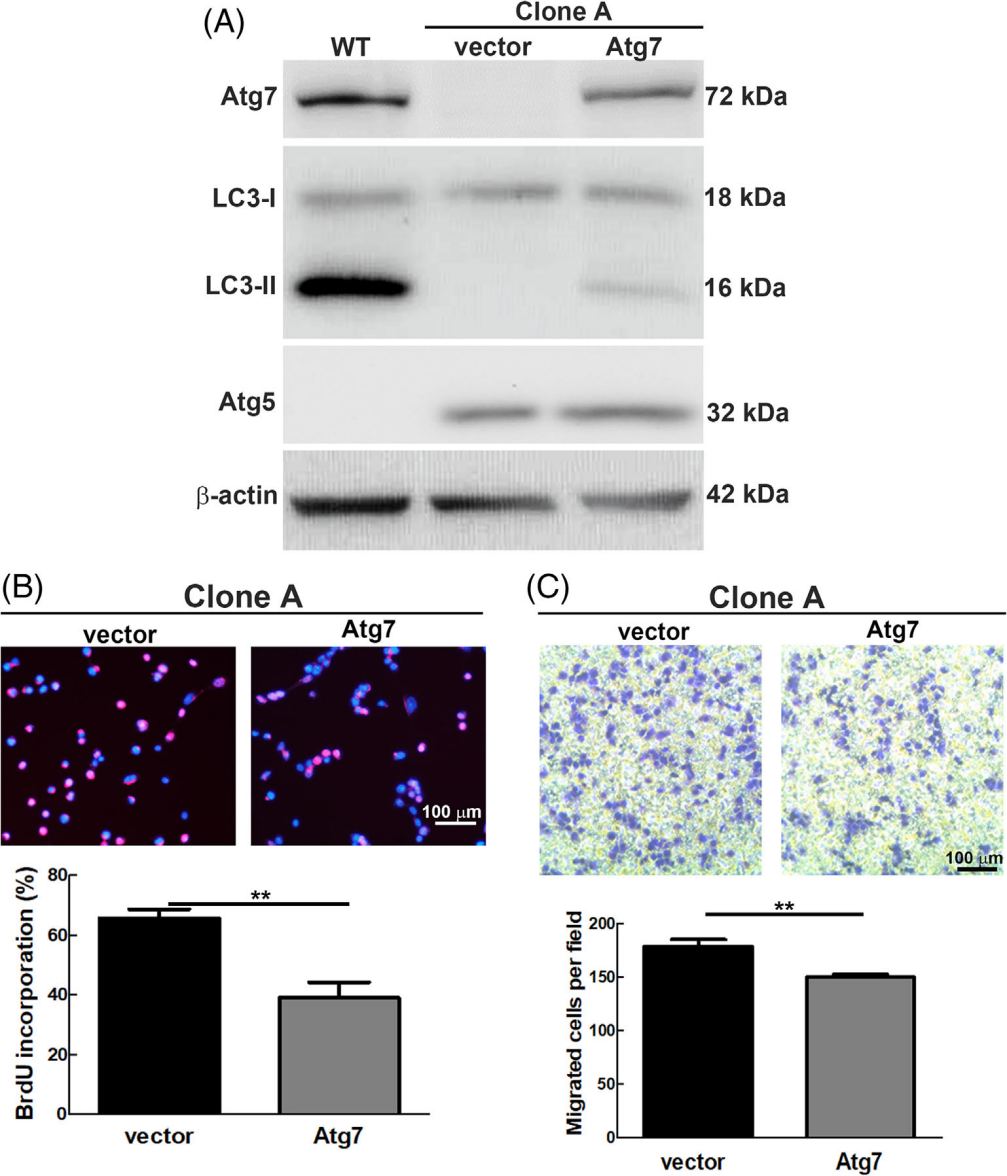
Rescuing autophagy deficiency of clone A (overexpression of Atg5 in Atg7^−/−^ MEF cells) suppressed cell proliferation and migration. Clone A cells were transfected with the plasmid pSF‐Atg7 or vector for 48 h. (A) The expression level of Atg7 and LC3 was measured by Western blotting using specific antibodies. WT MEF served as the positive control and β‐Actin was used as an internal control. (B) DNA synthesis was determined by BrdU (0.01 μg/mL) treatment for 20 min. Anti‐BrdU antibody and Hoechst were used to label proliferating cells and nuclei, respectively. Purple color represents proliferating cells, which were counted under three random fields under a fluorescent microscope at 200×. (C) Cells (1 × 10^5^) were seeded on the upper chamber in a 6‐well plate for 6 h. Migrated cells were stained with crystal violet and counted under three random fields under a light microscope at 100× magnification. The number of BrdU‐labeled cells and migrated cells between the vector and Atg7 groups were statistically analyzed by Student's t‐test. *P*‐values less than 0.05 were considered significant. **P < 0.01 (*p* = 0.001 and 0.0052 in panel B and C, respectively). Error bars represent SD.

We previously reported that tumor formation was suppressed when the HA‐Atg5 transgene was overexpressed to rescue the autophagic deficiency of MEF‐Atg5^−/−^‐Ras^val12^(M5R) cells, which induced robust tumor formation both in vitro and in vivo.^17^ Herein, we observed that clone A of Atg5‐overexpressed Atg7^−/−^ MEF cells induced larger tumor size compared to positive control M5R cells at day 3 p.i., followed by rapid regression (Figure 3B). We rescued the autophagy deficiency by overexpressing Atg7 transgene (pSF‐Atg7) in clone A MEF Atg7^−/−^ cells to clarify the role of Atg5 in the cell. The data showed that the protein levels of both Atg7 and LC3‐II genes in clone A were increased after transfection of the Atg7 transgene (Figure 4A), indicating autophagy activity was rescued. We then investigated the proliferation and migration of this Atg5‐overexpressed Atg7^−/−^ MEF cell line (Clone A) after autophagy activity was rescued by expressing the Atg7 transgene. Our data showed that both of cell proliferation and migration were suppressed by Atg5 when autophagy function was partially rescued (Figure 4B, C). In conclusion, our previous and current findings provide the compelling evidence that Atg5 functions either as a tumor suppressor in the cell with normal autophagy function or becomes a tumor inducer under autophagy‐deficient conditions during tumorigenesis.

### 3.5 Overexpression of Atg5 in Atg7^−/−^ MEF cells (clone A) leads to increased Wnt5a secretion and p‐JNK expression but decreased β‐catenin expression

**FIGURE 5:**
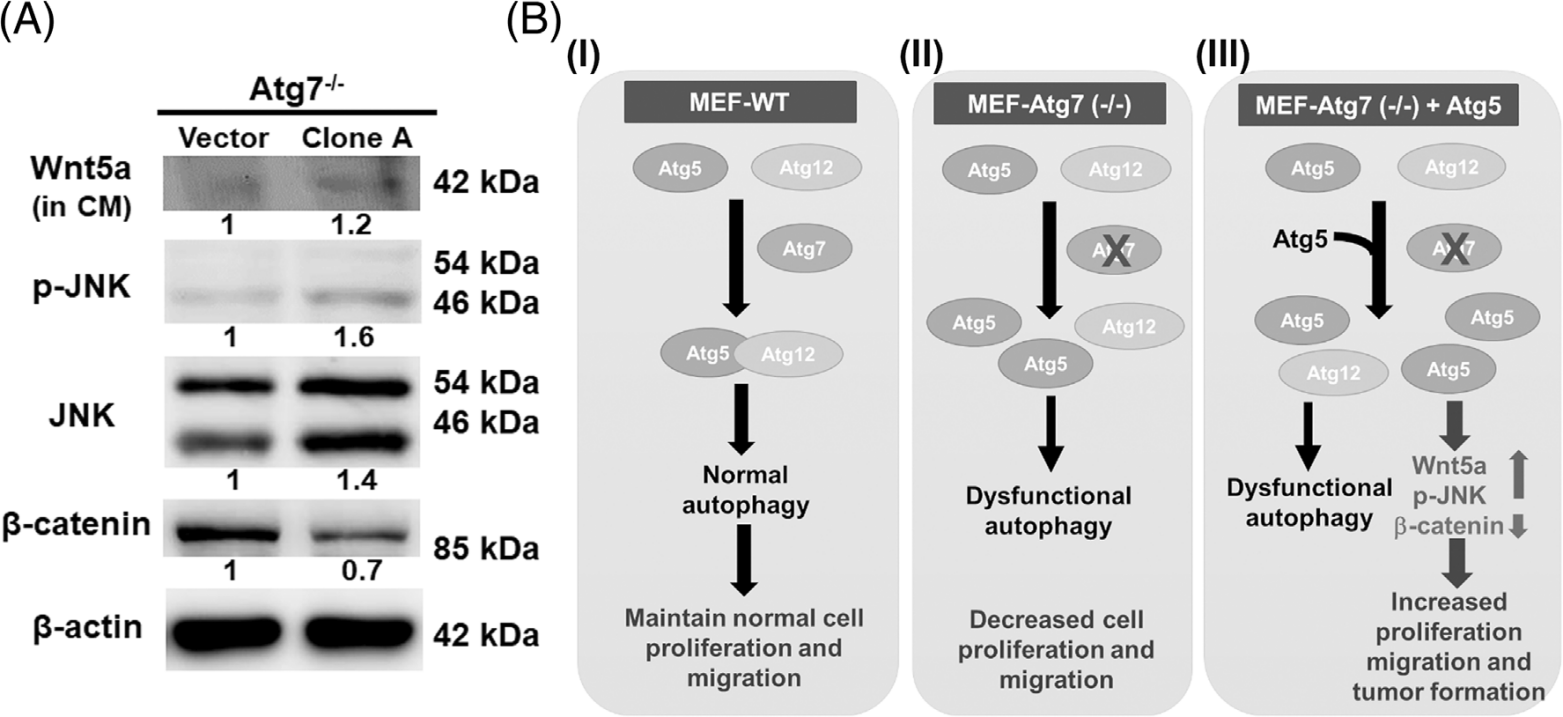
Overexpression of Atg5 in Atg7^−/−^ MEF cells (clone A) leads to increased Wnt5a secretion and p‐JNK expression but decreased β‐catenin expression. (A) Protein levels of β‐catenin, total JNK, and p‐JNK, as well as secreted Wnt5a of clone A were compared with those of vector control. Cell lysates were collected to measure the expression levels of β‐catenin, total JNK, and p‐JNK by Western blotting using specific antibodies. Conditioned media were collected and concentrated for detection of the secreted Wnt5a. β‐Actin was used as the internal control. (B) (I): In the cell with normal autophagy genes, Atg5 conjugates with Atg12 (Figure 1A) and the normal autophagy machinery proceeds, which suppresses cell proliferation and motility in vitro, and tumor formation in vivo. (II): In contrast, the cell with autophagy deficiency (Atg7^−/−^), Atg5 is not able to conjugate with Atg12 to carry out the processes of the normal autophagy machinery (Figures 1A and 2A). (III): Instead, overexpression of Atg5 activates the Wnt5a/p‐JNK/β‐catenin pathway to promote cell proliferation, migration in vitro, and temporal tumor formation in vivo.

We further explored the molecular mechanism involved in Atg5 promotion of tumorigenesis. Because the Atg5‐related signaling pathway participating in tumorigenesis remains unclear, we focused on the Wnt signaling pathway because autophagy and its crosstalk with the Wnt/β‐catenin axis has been reported.^20^ Wnt5a is a member of the noncanonical Wnt proteins and activates the β‐catenin‐independent noncanonical pathway to regulate cell polarity and movement through activation of the RhoA and Jun N‐terminal kinase (JNK) signaling cascades.^21^ It also inhibits the canonical Wnt pathway by promoting β‐catenin degradation in the presence of the ROR2 receptor.^22^ Our results showed increased phosphorylation JNK (p‐JNK) and decreased β‐catenin in clone‐A cells overexpressing Atg5 compared to the vector control cells by Western blotting (Figure 5A). Furthermore, we examined the Wnt5a in culture medium by trichloroacetic acid precipitation followed by Western analysis. The level of secreted Wnt5a in the concentrated condition medium (CM) of clone A was higher as compared to the vector control, indicating an elevation in Wnt5a secretion (Figure 5A). The tumorigenicity of Atg5 gene in Atg7^−/−^ MEF stable cells compared to vector control group have been demonstrated in Figure 2B–F and Figure 3A–C. Altogether, the Wnt5a/JNK/β‐catenin signaling pathway activated by overexpressing Atg5 under autophagy‐deficient conditions may contribute to increased cell proliferation, migration, and tumor formation in vitro or in vivo.

## 4 DISCUSSION

In this study, we demonstrated that abolishing autophagy essential genes (Atg5, Atg7, Atg9, and p62) consistently suppresses cell proliferation and migration (Figure 1A, E). Reduced cell proliferation was caused by decreased DNA synthesis not cell death (Figure 1C, D). We further reveal that the normal autophagy machinery plays pivotal roles in diverse cellular functions including cell proliferation and migration. MEF like NIH/3 T3 is an immortalized mouse fibroblast cell line that was isolated from a mouse embryo. Both cell lines have been proven to be easily transformed by DNA transfection of oncogenic factors such as *Ras* and *Myc* oncogenes. Therefore, we used it to clarify the role of Atg5 in tumorigenesis both in vitro and in vivo. Intriguingly, overexpression of Atg5 in autophagy‐deficient Atg7^−/−^ MEF stable clones significantly increased cell proliferation, colony formation, and cell migration in vitro (Figure 2), and transiently promoted tumor formation in vivo (Figure 3). These data reveal the oncogenic role of Atg5 under autophagy‐deficient conditions, and this oncogenic feature of Atg5 could be reversed by rescuing autophagy deficiency, indicating that normal autophagy suppresses the oncogenic role of Atg5 in tumorigenesis (Figure 4). Moreover, a mechanistic analysis revealed that the Wnt5a‐mediated JNK‐β‐catenin signaling pathway participates in Atg5 promoting tumorigenesis under autophagy‐deficient conditions. Altogether, the autophagy status determines the role of Atg5 in tumorigenesis (Figure 5 B [I–III]).

The role of Atg5 in autophagy‐related tumorigenesis remain contradictory. Atg5 has been shown to be a tumor suppressor including down‐regulation of Atg5 in colorectal cancer patients and early‐stage cutaneous melanoma.^23^ A low expression of LC3‐II representing low autophagy activity in melanoma was reported, and overexpression of Atg5 increased autophagy activity accompanied by decreased cell proliferation and colony formation.^23^ In the Atg5 gene knockout mouse model, the mice were normal at birth but died within 1 day.^24^ Another study reported that mosaic deletion of the Atg5 gene caused multiple liver tumors and Atg5‐deficient hepatocytes had a growth advantage over normal cells in vivo.^14^ Atg5 level is associated with prolonged disease‐free survival in breast cancers, implying a tumor‐suppressive role.^25^ A study on lung tumorigenesis revealed a prolonged overall survival and a reduced autophagic progression in autophagy‐deficient K*‐ras*‐Atg5^flox/flox^ mice; however, the tumor initiation was accelerated.^26^ A similar result was reported in patients with sporadic colorectal cancer, i.e., Atg5 was markedly downregulated. However, increased Atg5 expression was correlated with lymphovascular invasion.^27^ Based on big data prediction from the Cancer Genome Atlas (TCGA) database, the mRNA expression level of Atg5 was significant increase in stomach adenocarcinomna and compared with the normal part (Supplementary Figure S5). These findings imply that both of Atg5 and autophagy play dual roles in the tumorigenesis of diverse cancers.

Therefore, using autophagy‐deficient MEF stable cell lines (Atg7^−/−^) it is possible to verify the role of Atg5 in tumorigenesis. We established stable MEF clones overexpressing the Atg5 transgene in the background of Atg7 gene knockout (Atg7^−/‐^MEF), which showed increased cell proliferation, migration, and colony formation in vitro, and transient induction of tumor formation in vivo. In contrast, restoring the autophagic activity by expressing exogenous Atg7 in Atg7^−/−^ MEF cells shifted the role of Atg5 in cell proliferation and migration from promotion to suppression. This finding implies that the role of Atg5 in tumorigenesis is affected by the autophagy status within the cell.

However, subcutaneous inoculation of Atg5‐overexpressed Atg7^−/−^ MEF cells into NOD/SCID mice induced temporary tumor formation in the early stage of tumor formation. The tumors then regressed within 2 weeks. This result implies that Atg5 is a weak oncogenic factor under autophagy‐deficient conditions. Moreover, a single‐gene alteration, such as Atg5 under autophagy‐deficient conditions, might not be sufficient to induce a long‐term tumorigenic promoting effect capable of forming a tumor. Based on the two‐hit hypothesis proposed by Alfred G. Knudson in 1971,^28^ which was modified in 2001,^29^ the hits of multiple genetic mutations or gene activation may cause abnormal gene expression and lead to tumor initiation and progression. For instance, colorectal carcinoma arises from adenomatous polyps followed by progression to a benign neoplasm with a growth advantage. Five mutations linked to different genes are known to be involved in conversion of the polyp into the carcinoma.^30^ Our data showed the tumor regression under Atg5 overexpression accompanied with autophagy deficiency (Figure 3). Atg5 may act as the first “hit” to induce a benign tumor formation, however, the second “hit” to prolong tumor development and progression was not activated under autophagy‐deficient conditions.

We also noticed an interesting phenomenon: the average volume of tumors formed by clone A of Atg5‐overexpressed Atg7^−/−^ MEF cells was greater than that of M5R within 3 days (p.i.). This result might be due to the higher proliferation rate and the growth advantage of clone A of Atg5‐overexpressed Atg7^−/−^ MEF cells. Another possible reason is the individual differences of mice, since the variation was high at day 3. In this experiment, we did not evaluate the cell proliferation rate between clone A of Atg5‐overexpressed Atg7^−/−^ MEF cells and M5R. Thus, the difference between clone A of Atg5‐overexpressed Atg7^−/−^ MEF cells and M5R warrants further exploration.

A review paper reported that autophagy is tumor‐suppressive in the early stages of cancer and tumor‐promoting in established tumors.^31^ Interestingly, a heterozygous loss of the Atg5 gene results in further tumor progression and drug resistance response.^32^ However, in most studies examining the role of Atg5 in tumorigenesis, the status of autophagy activity was not verified. We are the first to reveal the dual roles of Atg5 in tumorigenesis dependent on the status of autophagy within the cell.

Wnt5a expression has been reported to be a potential target when Atg5 promotes tumorigenesis in an autophagy‐deficient background.^20^ Atg5 not only mediates a positive feedback loop between the Wnt signaling pathway and autophagy in melanoma cells,^33^ but also regulates cell polarity and movement through the activation of the RhoA and Jun N‐terminal kinase (JNK) signaling cascades.^21^ Our results showed decreased secretion of Wnt5a and its downstream JNK phosphorylation (p‐JNK) was increased in Atg5‐overexpressed Atg7^−/−^ MEF cells. The Wnt5a signaling pathway is well known to participate in various cellular processes, particularly in metastasis during tumorigenesis.^34^ Overexpression of Atg5 and the activation of p‐JNK might be responsible for cell migration. However, further clarification is needed to determine whether Wnt5a affects cell proliferation or migration, or both, in our model.

We also found that the β‐catenin protein level was decreased when Wnt5a secretion was increased in clone A of Atg5‐overexpressed Atg7^−/−^ MEF cells accompanied by increased cell proliferation. Regulation of cell proliferation by β‐catenin through interaction with transcription factors and nuclear receptors has been reported. According to its interacting partner, β‐catenin can induce or repress cell growth.^35^ β‐catenin in pancreatic and hepatocyte cells interacts with liver receptor homolog 1 (LRH‐1) to induce cyclin D1 and E1 expression, as well as to promote cell proliferation.^36^ In colorectal cancer, β‐catenin interacts with lymphoid enhancing factor (LEF‐1) and Krüppel‐like factor 4 (KLF4) to increase or decrease tumor cell proliferation, respectively.^37^ Moreover, Wnt5a suppresses β‐catenin to enhance hair follicle regeneration.^38^ In contrast, β‐catenin does not participate in epidermal proliferation and adhesion.^39^ In conclusion, our findings shed light on the biological regulation and relationship between Atg5 and the Wnt/β‐catenin axis under autophagy‐deficient conditions during tumorigenesis.

## 5 CONCLUSIONS

Herein, we demonstrate that autophagy deficiency leads to decreased cell proliferation and motility, indicating that autophagy plays a protective role in maintaining normal cell function^40^ (Figure 5 B [I]). We are the first to clarify the autophagy‐independent role of Atg5 in tumorigenesis both in vitro and in vivo. Overexpression of Atg5 under Ha‐*ras* oncogene‐induced autophagy^17^ or normal autophagy conditions suppressed cell proliferation and tumor formation to maintain cellular homeostasis. Without functional autophagy, Atg5 becomes a weak oncogenic factor that promotes initial tumor formation, but needs other oncogenic factors to prolong further tumor development.

## CONFLICT OF INTEREST STATEMENT

The authors declare no conflicts of interest.

## Supporting information


**Data S1.** Supplementary Information.
