# Supplementary material for: The role of Atg5 gene in tumorigenesis under autophagy deficiency conditions
Source: Kaohsiung J Med Sci. 2024 Jun 3;40(7):631–41. doi: 10.1002/kjm2.12853 (PMC11895631; doi:10.1002/kjm2.12853)
Supplement: Supplementary file 1 — Data S1. Supplementary Information. [file KJM2-40-631-s001.docx]

**Supplementary data**

**Supplementary Figure S1**


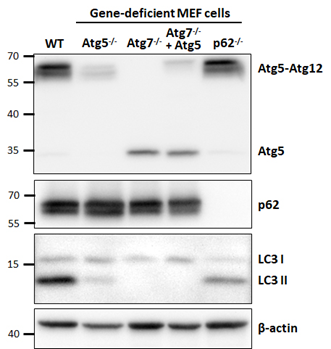


**Supplementary Figure S1**. **Protein expression of Atg-related gene in Autophagy deficient MEF cell lines.**

**Lane 1** showed the MEF-WT cell line with low Atg5 expression, resulting from the highest level of the Atg5-Atg12 conjugated complex. The covalent bond (-) within this complex prevents its denaturation during SDS-PAGE ^1^. **Lane 2** represents the MEF-Atg5(-/-) cell line, which was knocking down to silence Atg5 gene expression. This cell line exhibits no detectable Atg5 and a significant reduction in the Atg5-Atg12 conjugated complex. **Lane 3** showed Atg7 knockout in MEF cell (MEF-Atg7(-/-)), the expression of Atg5 is detectable, but there is no presence of Atg5-Atg12 conjugated complex. This absence confirms that the formation of the Atg5-Atg12 conjugated complex indeed requires Atg7 ^2^. **Lane 4** showed the result of restoring Atg5 expression in MEF-Atg7(-/-) cells. While Atg5 is expressed, the Atg5-Atg12 conjugated complex remains undetectable. The faint signal observed in the immunoblotting corresponds to GFP-Atg5 at 58 KDa (26 kDa + 32 KDa). **Lane 5** depicted the MEF-p62(-/-) cell line, which has a p62 knockout and exhibited low expression of Atg5 and elevated expression of the Atg5-Atg12 conjugated complex.

**Supplementary Figure S2**


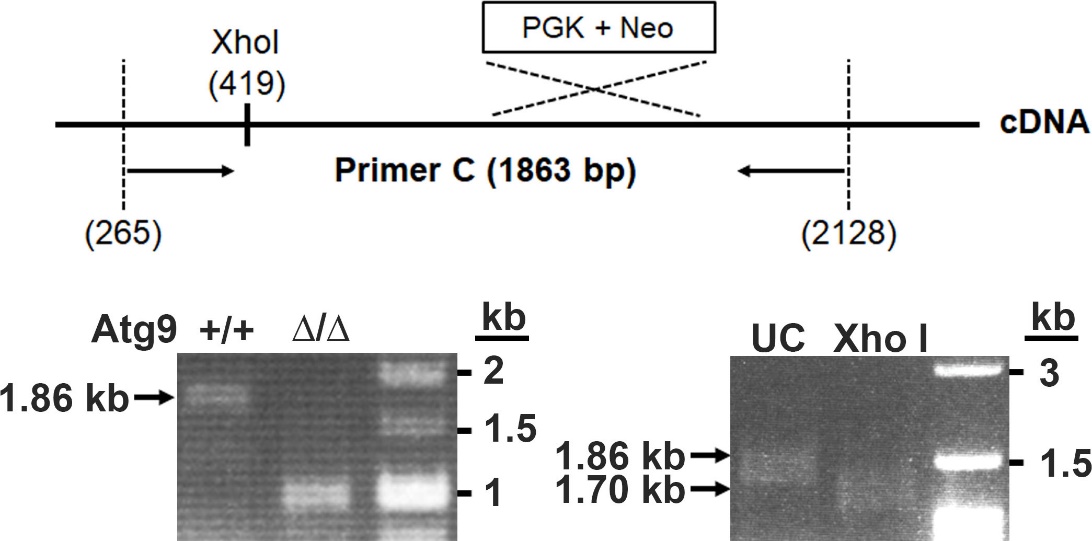


**Supplementary Figure S2. Schematic representation of cDNA of Atg9 gene in WT MEF.**

Atg9-/- MEF was constructed by the replacement of Atg9 exon 6 to exon 11 with the neomycin resistance gene. Total RNA extracted from WT and Atg9-/- MEFs was subjected to RT-PCR analysis. cDNA was amplified by primers C. PCR product of WT MEF was digested by *Xho* I.

**Supplementary Figure S3**

**
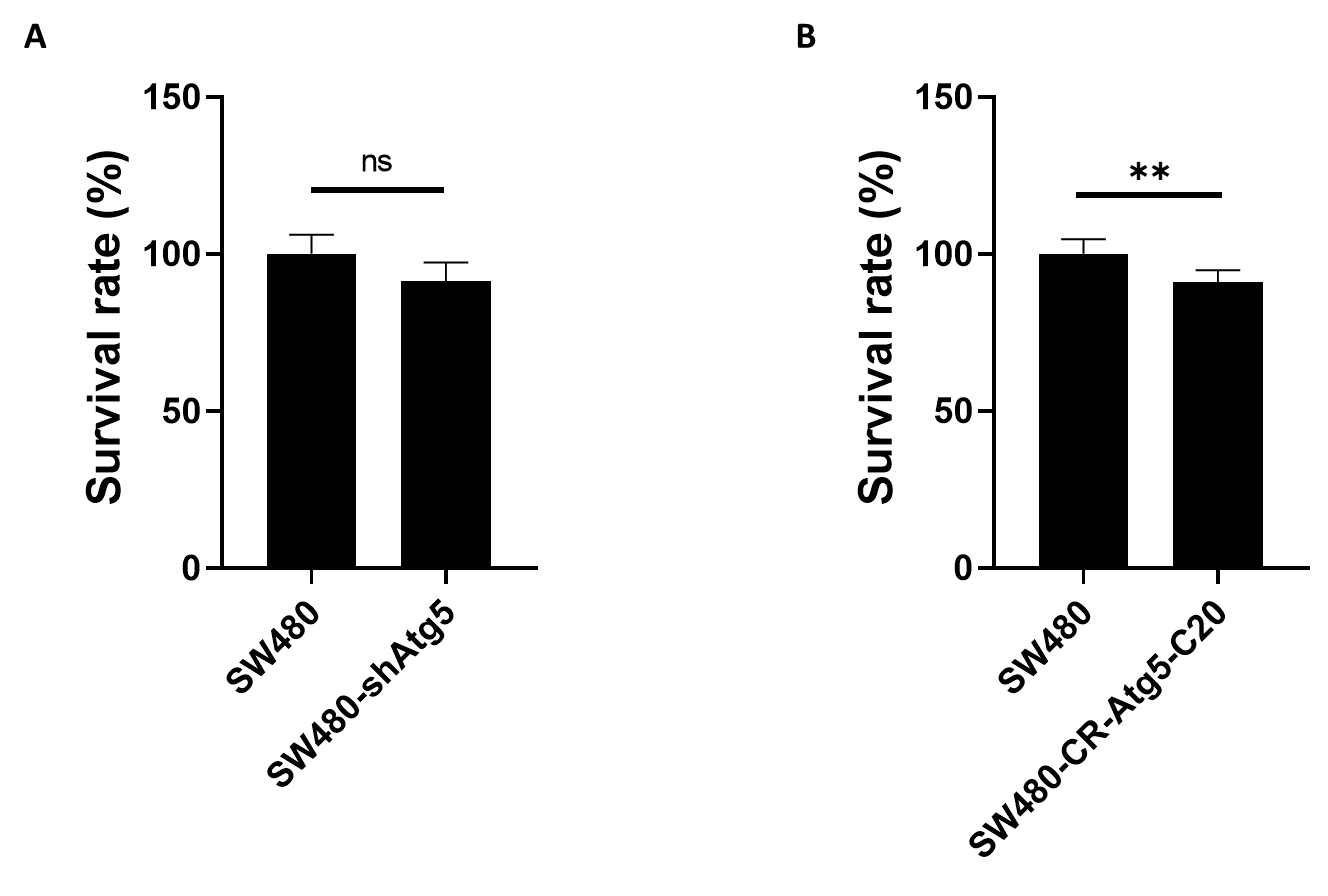
**

**Supplementary Figure S3. The effect of silencing and knockout Atg5 gene on the proliferation of SW480 cell lines**

(A) SW480 cells were treated with lentiviral shRNA to silence the Atg5 gene. (B) SW480 cells were cloned by CRISPR-Cas9 knockout Atg5 gene. SW480-CR-Atg5-C20 was the homologous recombination. Cell proliferation was determined by MTT assay at 570 nm wavelength for 48 h. Data were analyzed by Student’s t-test. Error bars represent mean ± SD. ns: no significant, **: p<0.01.

**Supplementary Figure S4**

**
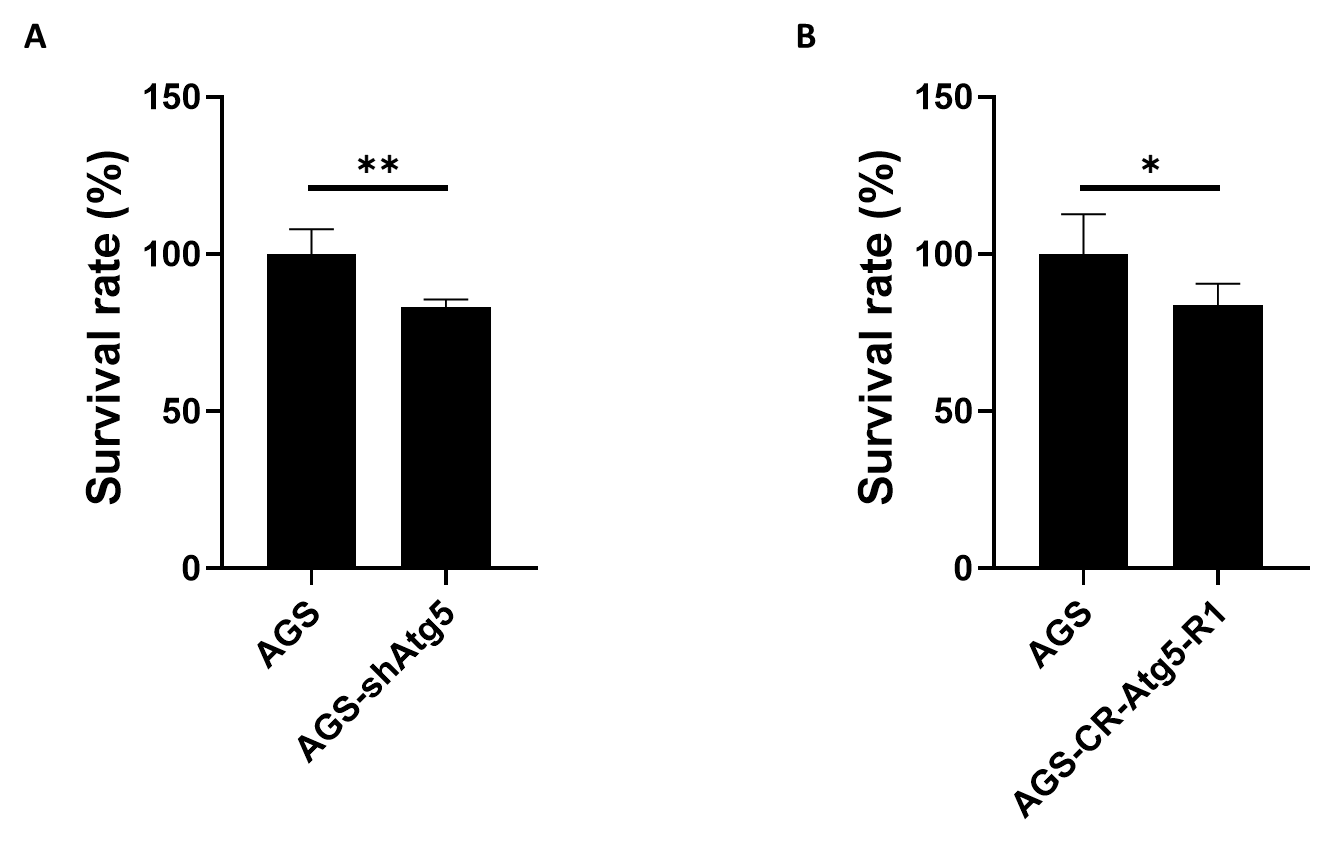
**

**Supplementary Figure S4. The effect of silencing and knockout Atg5 gene on the proliferation of AGS cell lines.**

(A) AGS cells were treated with lentiviral shRNA to silence the Atg5 gene. (B) AGS cells were cloned by the CRISPR-Cas9 knockout Atg5 gene. AGS-CR-Atg5-R1 was the homologous recombination. Cell proliferation was determined by MTT assay at 570 nm wavelength for 24 h. Data were analyzed by Student’s t-test. Error bars represent mean ± SD. *: p<0.05, **: p<0.01.

**Supplementary Figure S5**

**
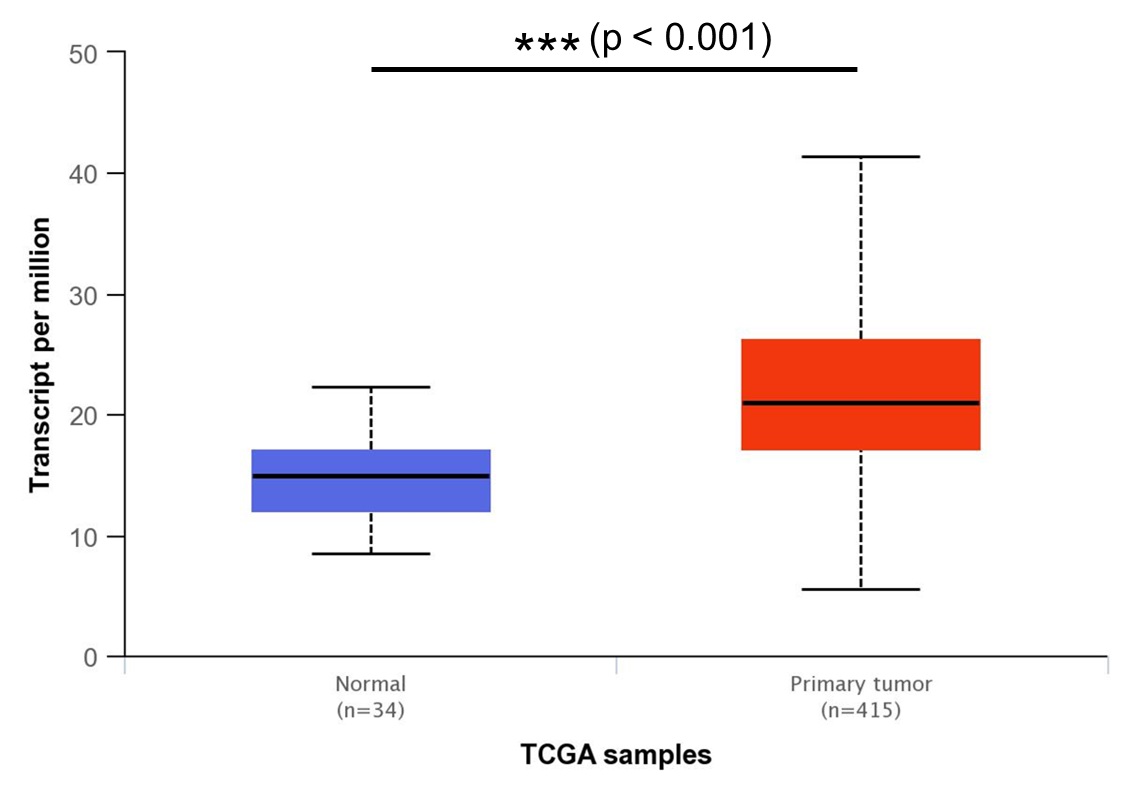
**

**Supplementary Figure S5. The expression level of Atg5 mRNA expression in** **stomach adenocarcinoma compared with normal clinical specimens from** **The Cancer Genome Atlas (TCGA) database.**

A total of 415 stomach adenocarcinoma and 34 normal clinical specimens from the TCGA database were analyzed for mRNA expression of Atg5. The transcript per million (TPM) expression levels of Atg5 were shown in the TCGA database, which significantly increased in tumor parts (***: p<0.001). The data was analyzed by the Student t-test.
